# A novel tissue engineered three-dimensional in vitro colorectal cancer model^☆^

**DOI:** 10.1016/j.actbio.2013.04.028

**Published:** 2013-08

**Authors:** Agata Nyga, Marilena Loizidou, Mark Emberton, Umber Cheema

**Affiliations:** a Centre for Nanotechnology and Regenerative Medicine, University College London, London, UK; b Division of Surgery and Interventional Science, University College London, London, UK; c Tissue Repair and Engineering Centre, Institute of Orthopaedics, Division of Surgery and Interventional Science, UCL Stanmore Campus, Brockley Hill, London HA7 4LP, UK

**Keywords:** Collagen I, Hypoxia, Plastic compression, Three-dimensional culture, Tumour microenvironment, Tumour invasion

## Abstract

The interactions of cancer cells within a solid mass with the surrounding reactive stroma are critical for growth and progression. The surrounding vasculature is recruited into the periphery of the growing tumour to supply cancer cells with nutrients and O_2_. This study focuses on developing a novel three-dimensional (3-D) in vitro biomimetic colorectal cancer model using colorectal cancer cells and connective tissue cells. The 3-D model comprises a dense artificial cancer mass, created by partial plastic compression of collagen type I containing HT29 colorectal cancer cells, nested in a non-dense collagen type I gel populated by fibroblasts and/or endothelial cells. HT29 cells within the dense mass proliferate slower than when cultured in a two-dimensional system. These cells form tumour spheroids which invade the surrounding matrix, away from the hypoxic conditions in the core of the construct, measured using real time O_2_ probes. This model is also characterized by the release of vascular endothelial growth factor (VEGF) by HT29 cells, mainly at the invading edge of the artificial cancer mass. This characterization is fundamental in establishing a reproducible, complex model that could be used to advance our understanding of cancer pathology and will facilitate therapeutic drug testing.

## 1 Introduction

The ability to grow cancer cells in vitro has informed much of our understanding of the biology of cancer. Its convenience and accessibility have made it a distinct alternative in many cases to in vivo animal experimentation. However, the simplicity of many of the models has also been their downfall, as they tend to be two-dimensional (2-D) and largely overlook host-related factors as determinants of tumour progression [1]. What has been missing is an in vitro model that has many of the known attributes of an in vivo cancer growing within the host environment. Some of these attributes are host related, while others are tumour related. Host-related factors might include defined three-dimensional (3-D) compartments, a range of tissue tonicities, appropriate stromal ratios (glycoproteins and proteoglycans) and relevant supporting cells in terms of both type and proportion. The tumour-related attributes necessary for a biomimetic model would ideally comprise differential cell proliferation, central hypoxia, compartment invasion and new vessel formation.

Most of the attempts to recapitulate these biomimetic properties have been achieved by shifting from 2-D culture studies to culturing cancer cells in 3-D scaffolds. Examples include the creation of multicellular tumour spheroids [2] and the generation of tumour colonies grown in tissue engineered structures [3]. 3-D models have, so far, been useful in furthering our understanding of many tumour-related processes, such as invasion and responsiveness to antitumour agents [4,5].

The 3-D models that have been described to date have incorporated a range of matrices, both natural and synthetic. The natural constituents typically comprise a laminin-rich extracellular matrix of collagen type I or hyaluronic acid [3,5]. Synthetic matrices include the use of polyethylene glycol (PEG), which is a biocompatible and biodegradable polymer used for a variety of tissue engineering applications. Synthetic matrices have the advantages of being easily modified to enhance biomimetic function, including the incorporation of bioactive peptides such as the integrin-binding motif RGD (Arg–Gly–Asp) [6], which can be achieved via fibrin stabilizing factor (factor XIII)-catalysed cross-linking [7], as well as engineered polymers with specific matrix metalloproteinase (MMP) sites for improved biodegradation [8]. Collagen type I hydrogels have been used more recently, with markedly different effects on cancer cell proliferation and cell signalling, namely the production of angiogenic growth factors. A limiting factor of these scaffolds is that they are mainly comprised of water, while to accurately mimic a tumour much denser collagen scaffolds need to be used [9].

As we engineer more biomimetic 3-D models we enhance our understanding and overcome current limitations, such as the use of cancer cell lines that form spheroids within a 3-D scaffold without a distinct stromal component [1]. These models are used for drug testing but are poor models for exploring innate tumour characteristics, given the absence of interactions with the stroma [10–13]. Most groups that have attempted to incorporate a cancer–stromal cell mix (usually fibroblasts) into a 3-D matrix [14] have done so without trying to recreate the tissue architecture seen in vivo. A recently described colorectal cancer model does place cancer spheroids in a suspension of fibroblasts housed in a collagen matrix [15]. This attempted to create a more biomimetic spatial configuration of a tumour surrounded by a reactive stroma, and this spatial mimicry resulted in a change in colorectal cancer cell morphology. Such biomimetic 3-D in vitro models represent a distinct advance on the traditional 2-D constructs [16].

This paper describes our attempt to narrow the gap between in vitro 3-D models and the in vivo scenario. Below we describe the creation of a 3-D in vitro colorectal cancer model that is biomimetic from a structural and spatial perspective and has the attributes of in vivo models. Validation of this model includes real time hypoxia measurements within the growing tumour.

## 2 Materials and methods

### 2.1 Cell culture

HT29 human colon adenocarcinoma cells were used to create the artificial cancer mass (ACM). For the surrounding tumour stroma we used either the mouse embryonic fibroblast cell line 3T3 (ECACC, Sigma-Aldrich, Poole, UK) or the human colon fibroblast cell line CF56, isolated from normal patient colon, as previously described [17]. (Informed consent was obtained from patients prior to surgery and the study was approved by the University College Hospital Independent Ethics Committee.) Briefly, primary human fibroblasts were isolated from areas of normal colon within a resected specimen from a patient with Duke’s B stage colorectal adenocarcinoma. Cells were extracted from macerated tissue adjacent to, but not part of, the cancer tissue. Antibody-coated magnetic beads were used to remove epithelial and endothelial cells; the remaining cells (β-actin positive) were propagated in culture and used between passages 4 and 12. Fibroblasts were used either alone or together with the human umbilical endothelial cell line HUVEC (ECACC). HT29, 3T3 and CF56 cells were cultured as 2-D monolayers in low glucose Dulbecco’s modified Eagle’s medium (DMEM) (1000 mg l^−1^ glucose, Sigma-Aldrich) supplemented with 10 vol.% foetal bovine serum (FBS) (First Link UK, Wolverhampton, UK), 100 U ml^−1^ penicillin and 100 μg ml^−1^ streptomycin (P/S) (Invitrogen, Paisley, UK). HUVEC were cultured in enriched endothelial cell growth medium (EECGM, PromoCell, Heidelberg, Germany) containing endothelial cell medium supplement (PromoCell), 10% FBS and P/S. Cells were cultured under aseptic conditions in a humidified atmosphere with 5% CO_2_ at 37 °C.

### 2.2 Engineering an in vitro biomimetic3-D cancer construct

ACMs were manufactured by seeding cancer cells in collagen type I hydrogels and applying plastic compression to increase the cell and matrix density. To prepare the collagen hydrogel containing HT29 cells collagen type I (rat tail collagen type I, 2.04 mg ml^−1^ in 0.6% acetic acid, First Link UK) was mixed with 10× concentrated minimum essential Eagle’s medium (MEM) (Invitrogen). The solution was neutralized in a drop-wise manner, first with 5 M and then 1 M NaOH, and assessed by a visible colour change from yellow to bright pink, indicating a pH of 7.3. At this stage the cancer cell suspension was added and the combination was gently mixed using a Pasteur pipette. The volume ratios of collagen type I:MEM:cell suspension were: 8:1:1. This cellular collagen solution was thoroughly mixed and transferred to a mould, then left to incubate for 30 min at room temperature, allowing the gel to set. Typically 4 ml of HT29 cell–gel solution was used.

Once set, the density of the HT29-containing cellular collagen gel (ACM) was increased by partial plastic compression [18]. The mould containing the gel was placed on a 165 μm steel mesh and a nylon mesh on absorbent paper. The gel was compressed under a 73.55 g load for 20 s. The load was removed and the gel was allowed to self-compress for a further 5 min. The gel was turned over and the process repeated.

The collagen density was assessed by comparing the wet and dry weights of the acellular gels that underwent none, partial or full compression. For full compression the load was applied to the gel for 5 min on both sides.

**Fig. 1:**
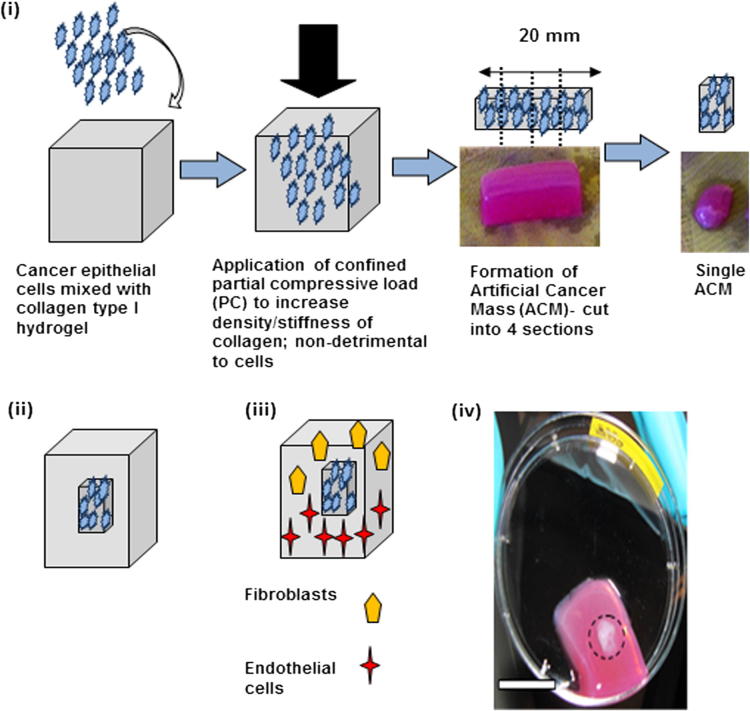
Schematic demonstrating construction of the 3-D in vitro cancer model. (i) Formation of an ACM. Partial compression of the cancer cell populated collagen hydrogel to increase the collagen and cell density. Division of the partially compressed ACM into appropriate final units. (ii) Insertion of the ACM into acellular collagen hydrogel. (iii) Insertion of the ACM into a cellular collagen hydrogel populated with fibroblasts and/or endothelial cells.

Partially compressed ACMs were cut into four pieces of approximately 5.5 × 10 × 10 mm using a sterile surgical blade. Two pieces were transferred to opposite sides of a rectangular plastic mould (75 × 25 × 15 mm). The mould was filled with 10 ml of collagen gel which was either acellular (collagen I:MEM ratio 9:1) or contained cells (collagen I:MEM:cell suspension ratio 8:1:1). In order to engineer a three cell type-containing gel, after the ACM was placed in the mould 5 ml of collagen gel containing fibroblasts was first added, followed by the addition of 5 ml of collagen gel containing endothelial cells. The uncompressed cellular gels (containing either fibroblasts or endothelial cells) were neutralized to pH 7.3 before being added to the mould following the method described above. A range of concentrations of fibroblasts and endothelial cells were tested (<2 × 10^6^ cells ml^−1^), with concentrations of 2.5 × 10^5^ cells ml^−1^ considered optimal. The whole process is schematically represented in Fig. 1.

### 2.3 Cancer cell proliferation in two and three dimensions

HT29 proliferation was assessed by the Alamar Blue assay, which measures chemical reduction of the dye, indicative of metabolic activity of mitochondrial enzymes, which is determined from the fluorescent signal at 570 nm. Partially compressed ACMs (1 ml) were placed in 6-well plates, covered in fully supplemented DMEM and incubated for up to 14 days. On days 1, 3, 5, 7 and 14 the medium was removed and 2 ml of 1:10 by volume Alamar Blue solution (AbD Serotec, Kidlington, UK) in phenol-free DMEM (Sigma-Aldrich) was added. Gels were incubated for 3 h at 37 °C on an orbital shaker at 4–5 g. Cell densities used for the ACM were 5 × 10^5^, 1 × 10^6^ and 2 × 10^6^ cells ml^−1^.

For 2-D culture cells were seeded in 6-well plates at 1 × 10^4^ and 2 × 10^4^ cells cm^−2^ and maintained at 37 °C in 5% CO_2_/air. Growth was assessed at the same time points as for the 3-D cultures, by Alamar Blue assay.

### 2.4 Cell morphology and migration within the 3-D cancer model

Cell morphology and migration in 3-D collagen constructs were determined by light microscopy of haematoxylin and eosin (H&E) stained sections. Constructs were fixed for 2.5 h in 10% neutral buffered formalin, processed in an automated tissue processor (Tissue-TEK® VIP, Sakura Fineterk, Torrance, CA) and embedded in paraffin. Sections were cut at 10 μm and placed in an oven at 60 °C overnight. Sections were rehydrated through xylene and 100%, 90% and 70% alcohol solutions, stained with haematoxylin for 5 min, immersed in blueing solution and stained with eosin for a further 5 min. Finally, sections were taken back through 70%, 90% and 100% alcohol solutions and xylene and mounted in DPX mountant (Sigma) for viewing.

### 2.5 Hypoxia

**Fig. 2:**
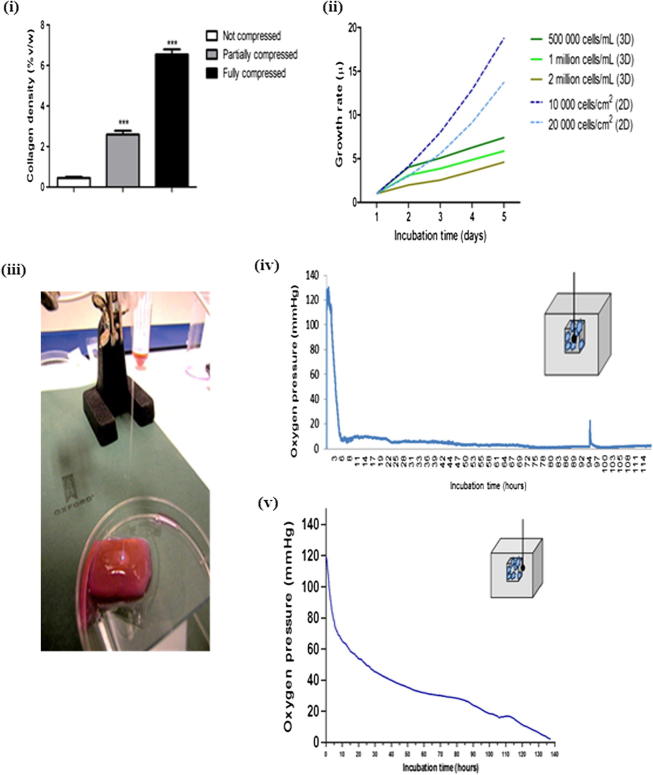
Characteristics of the collagen type I gel and ACM. (i) Collagen density of the acellular gel after partial and full compression. (ii) Growth rate of HT29 cells in 3-D collagen gels and on cell culture plates (2-D) obtained from the metabolic activity measured by Alamar Blue assay. (iii) Representation of the 3-D tumour model with inserted optic fibre probe measuring the oxygen level (light), (iv, v) Oxygen levels in the ACM populated with HT29 and surrounded by acellular collagen type I measured by tissue oxygenation monitoring: (iv) oxygen level in the core of the ACM and (v) oxygen level at the interface between the ACM and surrounding gel.

Oxygen levels were measured in 3-D constructs comprising the ACM nested in acellular collagen using an Oxylite 4000 tissue oxygenation monitoring system (Oxford Optronix, Oxford, UK). A fibre optic probe was inserted into the 3-D construct either deep within the ACM or at the ACM/“stroma” interface (Fig. 2iv and v). Oxygen levels were measured for up to 140 h in open cultures under routine conditions (Fig. 2iii–v). The tip of the sensor probe (280 μm diameter) incorporates an oxygen-sensitive luminescent probe within an oxygen permeable matrix, where the luminescence is quenched in the presence of molecular oxygen, thus the emission lifetime is longer at lower oxygen concentrations. Calibration of the probe, which is accurate to 0.7 mmHg, is reliant on correlation of the luminescence lifetime (rather than intensity) versus the oxygen concentration [19]. This method results in a stable calibrated response such that each probe can be used for up to 7 days at the slowest sampling rate. After each experiment the probe was checked against the external medium to confirm that there was no drift in the response. The fibre optic probes were used in conjunction with an OxyLab pO2 E™ system coupled to an A/D converter (12 bit) and the results were recorded on an IBM PC computer using Labview. The results are presented as partial pressure values, i.e. *p*_O2_ in mmHg (e.g. 7.6 mmHg corresponds to 1%).

In addition, hypoxia was verified by staining with pimonidazole, according to the manufacturer’s instructions. Pimonidazole is activated and forms protein adducts in cells at oxygen partial pressures <10 mmHg. Before fixing the 3-D constructs were incubated for 2 h in 100 μM pimonidazole hydrochloride (Hypoxyprobe™, Natural Pharmacia International, Burlington, VT) with agitation at 37 °C. Constructs were fixed and processed as standard. 4 μm sections were peroxidized for 5 min in 3% H_2_O_2_ (Calbiochem Milipore, Watford, UK) and incubated for 20 min at 55 °C in citrate buffer (pH 6). Sections were washed in rinse buffer and incubated for 5 min in blocking buffer (1% bovine serum albumin (BSA) in Tris-buffered saline (TBS)). After further washing the primary antibody fluorescein isothiocyanate (FITC)-labelled MAb1 (1:50 in primary antibody diluent, Hypoxyprobe™) was applied for 1 h at room temperature. Sections were washed and incubated with secondary rabbit anti-FITC IgG (1:50 in phosphate-buffered saline (PBS)) for 30 min at room temperature. Sections were then peroxidized for 10 min in 3,3′-diaminobenzidine (DAB), washed in distilled water and rinse buffer and counterstained with haematoxylin for 1 min. Dehydrated samples were mounted with DPX and assessed under a light microscope (Optika, Italy) using digital imaging system Infinity 2 (Digital Imaging Systems, Bourne End, UK).

### 2.6 VEGF production

Indirect immunofluorescent staining was performed to investigate the presence of VEGF. 4 μm sections (prepared as above) were microwaved in citrate buffer (pH 6) at 800 W, followed by three 10 min washes in PBS. Sections were treated with permeabilization solution (Millipore, Watford, UK) for 15 min and blocking buffer for a further 15 min. Slides were incubated with rabbit polyclonal anti-VEGF (Santa Cruz Biotechnology, Santa Cruz, CA) overnight at 4 °C in a humidified chamber. Both antibodies were diluted 1:50 in primary antibody diluent (Abcam). Sections were then washed three times in PBS and incubated with goat Alexa Fluor 546-labelled anti-rabbit antibody (orange fluorescence, 1:100 in PBS) for 2 h in the dark at room temperature. After incubation the sections were washed in PBS (3 × 10 min) and mounted in Vectashield/DAPI. Slides were assessed using a fluorescence microscope (Olympus BX61, Southend-on-Sea, UK) using Cell^F^ fluorescence imaging software (Olympus).

### 2.7 Statistical analysis

The data were parametric and treated by one-way ANOVA followed by post hoc analysis.

## 3 Results

### 3.1 Collagen density

Partial plastic compression of acellular collagen I hydrogels resulted in a six times increase in collagen density to 12 mg ml^−1^ (2.6%). The surrounding collagen hydrogel remained uncompressed with a collagen density of 2.04 mg ml^−1^ (0.25%). Complete or full compression of acellular hydrogels resulted in a 16 times increase, giving a collagen concentration of 33 mg ml^−1^ (7.2%) (Fig. 2i).

### 3.2 Cancer cell proliferation in three dimensions

Proliferation of HT29 cells was assessed in both two and three dimensions. HT29 were seeded in a partially compressed collagen I gel (initial cell densities 5 × 10^5^, 1 × 10^6^ and 2 × 10^6^ cells ml^−1^) or grown on a tissue culture plate (cell densities 1 × 10^4^ and 2 × 10^4^ cells cm^−2^). Growth was assessed by Alamar Blue assay.

The rate of growth was similar for cells in both two and three dimension cultures up to day 2. Between 3 and 14 days 2-D cultured cells continued growing in an exponential fashion, while 3-D cultures were considerably slower, following a steady rate of growth (Fig. 2ii). From repeat experiments over a wider range of HT29 cell densities for ACM construction the optimal density was determined to be 1.6 × 10^6^ cells ml^−1^, and this was used in experiments incorporating further cell types described below.

### 3.3 Cell morphology and migration in the 3-D cancer model

**Fig. 3:**
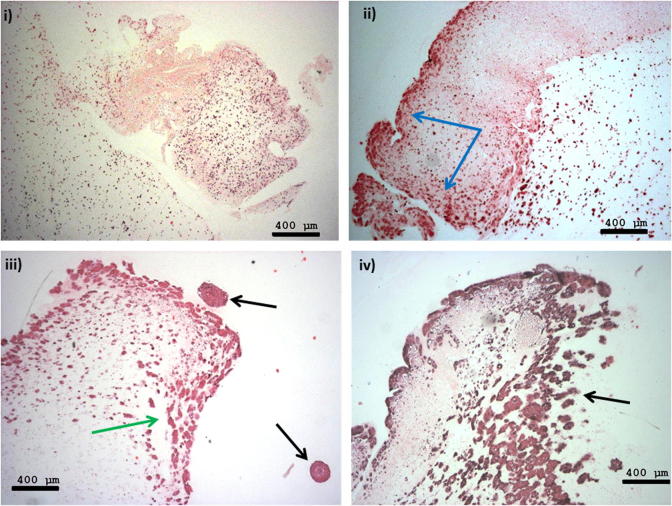
Morphology of HT29 colorectal cancer cells (2,000,000 cells ml^−1^) in an ACM situated within an uncompressed acellular collagen gel (H&E staining). (i) Day 1 showing an equal distribution of cells within the ACM; (ii) day 7 showing accumulation of cells at the edge of the ACM (blue arrows); (iii) day 14 showing formation of cell spheroids (green arrows) and migration into the surrounding matrix (black arrows); (iv) day 21 showing escape of the cell spheroids into the surrounding matrix (black arrow). Scale bar 400 μm.

**Fig. 4:**
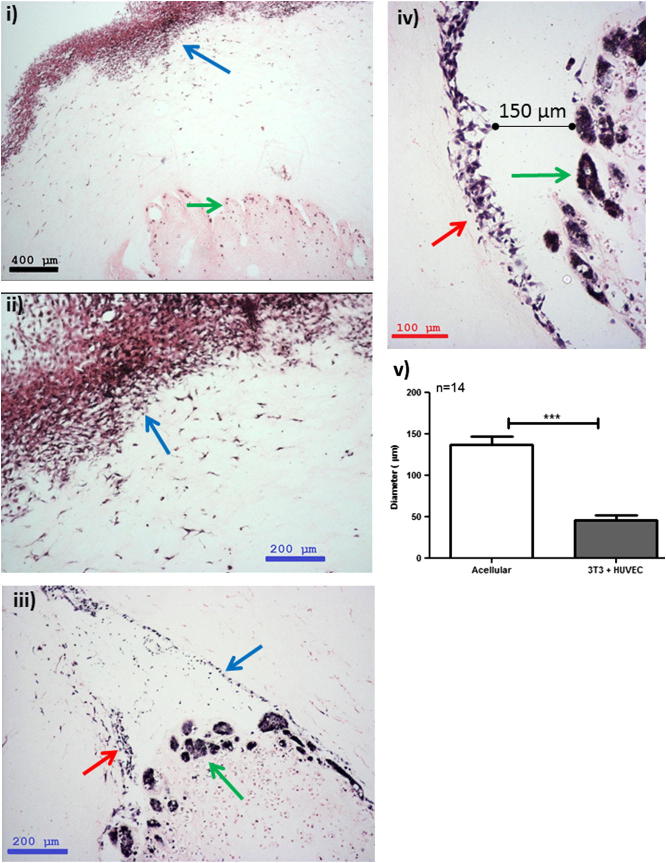
Morphology of HT29 colorectal cancer cells (500,000 cells ml^−1^) in an ACM situated within a collagen gel. (i, ii) The surrounding collagen was populated by 3T3 cells (blue arrow, 250 000 cells ml^−1^) and H&E staining was performed on day 14. (iii–v) The surrounding collagen was also populated by 3T3 cells together with HUVEC (250 000 cells ml^−1^) and H&E staining was performed on day 21, showing HT29 cell spheroids (green arrows) with sizes smaller than in Fig. 3 and detachment of the ACM from the surrounding matrix (by 150 μm) lined by 3T3 cells (blue arrow) and HUVEC (red arrow) into the surrounding matrix. Scale bars: black, 400 μm; blue, 200 μm; red, 100 μm.

3-D constructs were engineered at different levels of complexity. The simplest constructs were designed with ACMs containing two different seeded HT29 cell densities, either 5 × 10^5^ or 2 × 10^6^ cells/ml, nested in non-compressed acellular collagen I gels. These were cultured for up to 21 days. Within 24 h HT29 cancer cells were observed to be evenly spread throughout the dense collagen of the ACM (Fig. 3i). Within 7 days cell spheroids were formed within the ACM (Fig. 3ii), which appeared to move to the edge of the dense ACM and to migrate into the non-dense surrounding collagen by 14 days (Fig. 3iii). This migration of cell spheroids into the surrounding uncompressed collagen was observed up until day 21 (Fig. 3iv). The more complex 3-D constructs incorporated more cell types (fibroblasts and/or endothelial cells) within the non-compressed collagen hydrogel surrounding the ACM. Addition of 3T3 (or CF56) fibroblasts to the surrounding matrix induced a decrease in the volume of the whole collagen construct, due to fibroblast-generated collagen contraction, a known phenomenon in collagen hydrogels [20,21]. The addition of 3T3 fibroblasts also appeared to result in a decrease in the proliferation rate of cancer cells within the ACM. Spheroids/cancer foci on day 21 were smaller in more complex constructs with cellular stromal surrounds, and averaged 45 μm (. 4iii), compared with those with acellular stromal surrounds, where the spheroid size was found to be three times as large at 145 μm (*p* < 0.001) (Figs. 3iv and 4v). 3T3 fibroblasts showed migration towards the outer edge of the construct from day 1, with fibroblast multilayers observed by day 14 (Fig. 4i and ii). Interestingly, when endothelial cells (HUVECs) were added to the surrounding collagen together with 3T3 cells limited contraction of the collagen hydrogel was observed, suggesting partial opposition to the contraction induced by 3T3 cells alone. Non-cancer cells situated in the surrounding uncompressed collagen hydrogel migrated in two directions, towards the outer edge of the construct and also towards the ACM. The contraction events could at times cause separation of the dense ACM from the surrounding collagen I matrix, as demonstrated on day 21 in the 3-D constructs (Fig. 4iii and v). Separation of the dense ACM could be also affected by collagen processing for histological analysis.

### 3.4 Hypoxia within the 3-D cancer model

Oxygen levels were monitored in the 3-D constructs using fibre optic probes, within the ACM core and at the interface of the ACM and the surrounding uncompressed acellular collagen, for up to 6 days. Oxygen levels within the ACM showed a dramatic drop from 125 to around 10 mmHg within the first 4–5 h. The oxygen level then fluctuated between 2 and 10 mmHg (0.3–1.3% oxygen) over the following 112 h (Fig. 2iv). This level is considered equivalent to pathological hypoxia. The oxygen tension at the boundary between the ACM and uncompressed collagen was characterized by a slower drop from 130 to around 70 mmHg in the first 4 h. It was followed by a steady gradual drop to around 20 mmHg in the next 100 h, which reached around 5 mmHg at 134 h (Fig. 2v).

**Fig. 5:**
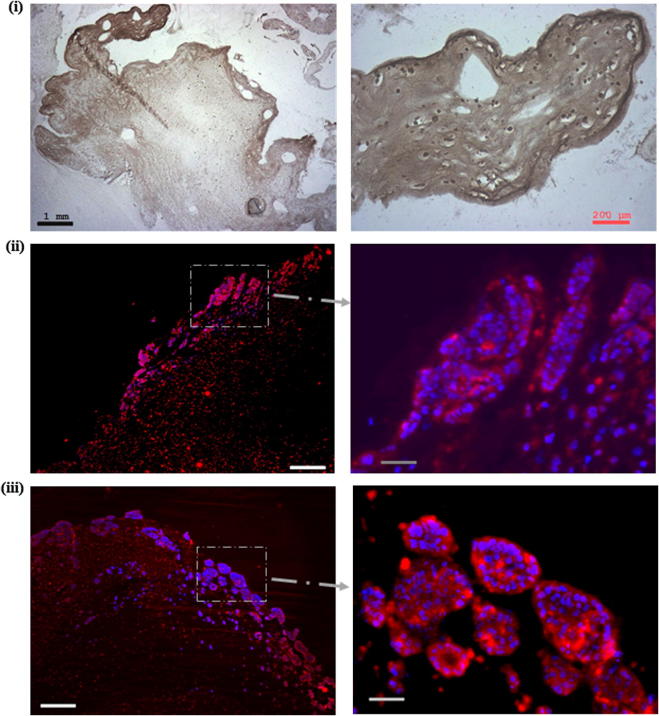
Immunostaining for hypoxia and VEGF release in ACM surrounded by acellular gel. (i) Hypoxia was confirmed by positive binding of antibody (brown) against pimonidazole, an exogenous hypoxia marker, and can be observed on day 14. Scale bar: black 1 mm; red, 200 μm. (ii, iii) VEGF release was confirmed by anti-VEGF staining (red), with DAPI counterstaining (blue) on (ii) day 14 and (iii) day 21. Scale bars correspond to 1 mm and 200 μm for the main display and higher magnification, respectively.

Hypoxia within the ACM was confirmed by positive staining with pimonidazole (HT29 concentration 1.6 × 10^6^ cells ml^−1^). This was evident in the ACM on days 7 and 14, with no positive staining in the outside non-dense gel populated by CF56 cells and HUVEC (both at a density of 2.5 × 10^5^ cells ml^−1^) (Fig. 5i).

### 3.5 Angiogenic response in the 3-D cancer model: VEGF production

VEGF expression was determined by immunofluorescence. Positive staining was observed in cancer cells throughout the ACM (surrounded by acellular collagen) from day 7. On days 14 (Fig. 5ii) and 21 (Fig. 5iii) VEGF expression was clearly evident in cancer cell spheroids/foci.

Production of VEGF was greater in cultures where the ACM was surrounded by a cellular hydrogel (3T3 without or with HUVEC).

## 4 Discussion

### 4.1 Summary of results

We have created a 3-D cancer model from HT29 cell lines characterized by a dense artificial cancer mass surrounded by a non-dense matrix populated by fibroblasts and endothelial cells. Spheroid size was directly related to whether or not the surrounding stromal environment was acellular or populated with cells. The additional biomimetic properties of the ACM permitted multidirectional migration and growth into the surrounding “stromal” component. Hypoxia within the tumour was also demonstrated, and confirmation of VEGF expression by cancer cells was considered a marker of the initiation of angiogenesis.

### 4.2 Methodological considerations

The 3-D tumour model comprised distinct regions, in terms of both the cell composition and collagen density, in a configuration which was spatially relevant to solid tumour growth in a tissue. Integration of the two components, which differed significantly in their collagen composition, was difficult in early cultures. By optimizing cell densities we were able to, in part, overcome this challenge of integration by ensuring the outer collagen hydrogel, stroma equivalent, contained enough cells to re-model the matrix.

Accurate real time O_2_ measurements in tumours in vivo are a challenge, but it is well documented that the hypoxic core of tumours quickly becomes necrotic due to the lack of an oxygen supply. Using probes to measure and assess O_2_ tension in our ACM and the surrounding “stroma” we were able to draw comparisons with the tumour scenario in vivo. In future these measurements will be used to assess tumour growth and invasion in this model.

The HT29 cell line is a spontaneously immortalized colorectal adenocarcinoma line, widely used in laboratory modelling. Its described mutations, e.g. *P53*, are relevant to colorectal oncogenesis and it exhibits moderate differentiation (glandular organization) and invasion in in vivo models. Invasion by HT29 cells from the ACM into the surrounding matrix (both acellular and cellular) was assessed using histology. The observed features included approximate glandular formation by cancer cells concentrated at the outer edges of the ACM, which often appeared to break away from the main ACM body and invade the surrounding hydrogel. This picture is very similar to that found in human biopsies of colorectal cancer.

### 4.3 The use of plastic compression to control cancer mass density

Advances have been made in increasing the density of biomimetic collagen scaffolds in a controlled manner. These processes include increasing the concentration of collagen in acid solution and/or the physical expulsion of excess fluid by means of compression. In contrast, methods that have been used to increase the concentration of collagen rely principally upon techniques that promote evaporation [22]. Other methods that have been reported include reverse dialysis [23], continuous injection of low concentration collagen solutions into glass microchambers [24], or a combination of the two [25]. These methods have a limitation on the volumes produced (reverse dialysis) and the formation of concentration gradients (injection method).

The application of a controlled load to plastically compress (PC) standard collagen hydrogels to expel excess fluid and increase the collagen density has become particularly useful in the field of tissue engineering [18]. Applications of this PC technology are useful for in vitro tissue modelling, as cell viability is retained in cells embedded within scaffolds undergoing PC, and the density increase (both for matrix and cells) is controllable and results in higher biomimetic densities. Using partial plastic compression we increased collagen densities by up to 10 times. We now aim to further our progress in this area by engineering ACM densities of 8–11% and even higher. The goal of biomimetic tissue engineering is to control the scaffold architecture such that it is truly biomimetic, we therefore believe that engineering the density is a critical first step.

### 4.4 Comparison with other studies

Our manipulation of collagen density using PC is the first use of this technology for 3-D tumour culture. It allowed us, in part, to culture cells at a more biomimetic tissue density. We controlled collagen density using a partial plastic compression method, which resulted in a final collagen percentage of up to 4% [26]. This high density collagen was surrounded by a non-compressed gel (0.25% collagen concentration). The use of intact native, fibrillar collagen as a 3-D matrix in which to grow cells is hugely beneficial in mimicking the native cell–matrix interactions found in vivo. Cell–matrix interactions are known to affect cell proliferation, differentiation and cell signalling [27]. Use of the plastic compression method conserved the fibrillar collagen structure and increased the collagen density to that approaching the levels found in vivo, with limited loss of viability [18]. Applying the plastic compression technique we have engineered dense tumour masses which are biomimetic.

Growth rates of HT29 cancer cells in our 3-D model were in line with the growth rates seen in vivo. After 7 days the HT29 cells displayed significantly lower growth rates than those seen in 2-D cultures. This differential growth pattern has been previously described. Glioma cell lines cultured in Matrigel and chitosan scaffolds exhibited slower growth rates than those seen in 2-D systems [28]. The slower growth rates seen in 3-D models more closely resemble those seen in vivo, as a consequence of the restraints on energy availability within a solid structure and its given surface area.

Our model structure allowed us to observe aspects of growth and migration hitherto not reported in other systems. The initiation of formation of HT29 spheroids was seen on day 7. By day 14 these spheroids had migrated outside the ACM into the non-compressed surrounding hydrogel. The stiffness of the matrix influences the formation of spheroids [7]. Softer PEG hydrogels (1.5%) tend to form more irregular and scattered spheroids. Increasing the density of the hydrogel (2.5%) forces the creation of more compact and dense spheroids, once again mimicking what we find in vivo*.* The mechanism for this is likely to involve changes in integrin binding sites, which, in turn, promote focal adhesion assembly leading to increased growth factor-dependent ERK activation [29].

The other novel attributes of our system relate to the incorporation of fibroblasts in the non-dense collagen gel surrounding the ACM, mimicking the natural tumour stroma. Fibroblast seeding caused collagen matrix contraction and, thus, a decrease in the volume of the collagen construct within the first 3 days. A similar phenomenon was observed when fibroblasts were co-cultured with human breast tumour spheroids in a collagen type I gel [14]. This fibroblast-mediated contraction of the whole system further inhibited the access of cancer cells in the ACM to both nutrients and oxygen. This effect may further serve to align growth rates to those observed in vivo. The mechanism by which this occurs is known [30]: fibroblasts release proteinases, such as MMPs, that degrade collagen and disrupt the formation of co-units by other cells. One of the most exciting findings from this model has been the dependence of spheroid size on the stromal component (Fig. 4). Where cells are present in the stromal component the spheroid size was limited, in fact three times smaller than when no cells were present in the stromal surround. It is likely that paracrine signalling by stromal cells limits spheroid size. Cell–cell interactions between a cancer mass and the surrounding stroma influence tumour growth and metastasis, and it is intriguing to see this recapitulated in our biomimetic model. Further analysis is necessary to understand this interaction, but this model provides an ideal platform for research in this area.

Contraction of the system was limited by the addition of endothelial cells to the fibroblasts-seeded stromal component. On day 21 these stromal cells were found not only on the outside edge of the construct but also in close proximity to the ACM. However, the ACM started to detach from the surrounding collagen, which could be caused by the proteinases released by surrounding cells. We are currently investigating why the fibroblasts essentially go one of two ways: a portion of the fibroblasts appears to be attracted to the ACM, while the other migrates towards the outer edge, towards the highest oxygen and nutrient levels. The detaching ACM was also characterized by the presence of tumour spheroids. These morphological changes in cancer cells indicate their potential invasiveness by forming spheroids that migrate into different regions of the system.

The incorporation and measurable tracking of hypoxia within our 3-D system is novel. Hypoxia is one of the important drivers of tumour growth. Our system was associated with oxygen depletion in the core from 125–130 to around 10 mmHg within the first 4 h. The changes at the outer edge of the ACM were less dramatic in both rate and degree. Within the first 10 h oxygen levels dropped to below 60 mmHg, and after 90 min to around 20 mmHg. Pimonidazole staining confirmed the hypoxic regions at the edges of the ACM, to which most cancers cells had migrated. Other groups have documented hypoxia using pimonidazole staining in multicellular spheroids cultured in nano-imprinted cycloolefin resinous scaffolds [31].

The effect of hypoxia on tumour-initiated angiogenesis was seen in our model. Positive VEGF immunofluorescence staining was seen in ACM on days 7, 14 and 21. On day 7 VEGF was found throughout the ACM, however, from day 14 its release was mainly found around newly formed tumour spheroids. In the model incorporating both fibroblasts and endothelial cells VEGF release was observed within the first 24 h. Moreover, it was present in both ACM and the surrounding collagen. This confirms that VEGF was released by both the growing tumour cells and the surrounding supporting cells. VEGF production by the stromal cells also indicates that the fibroblasts and endothelial cells were in a hypoxic environment, probably created by cell consumption of oxygen within this stromal compartment.

### 4.5 Implications of the research

Having a 3-D model with biomimetic properties raises new and important possibilities.

Firstly, a model which closely approximates our current understanding of the spatial and architectural nature of tumours provides us with a platform to further our understanding of the importance and implications of these interdependent structures. The tumour–stromal interactions that we have documented concur closely with those described in vivo*.*

The second point relates to developing novel therapies, whether they are physical or chemical. When these 3-D models exhibit hypoxia gradients they may be used to refine new radiotherapy techniques, given that hypoxia is the greatest impediment to successful radiotherapy. Chemotherapeutic agents and biological modifying agents could be assessed during the early target verification phase in a more meaningful manner with the 3-D models that we have described, as they are much closer to the real targets that they are seeking to modify. The complexity of the 3-D in vitro models will allow the mechanisms by which novel therapies may work to be explored.

Third, 3-D models give the opportunity to gain an insight into the way in which tumours are currently imaged in clinical practice. Multiparametric magnetic resonance imaging uses sequences that measure cell density by virtue of the Brownian motion of protons within the cancer cells and within the extracellular space. These indicate the diffusion sequences and are becoming the mainstay of cancer diagnosis and monitoring in most organ systems. Our 3-D model is amenable to sequential imaging, a process that will allow us to understand the biological changes that trigger these different signals.

Fourth, we have described the basis of a platform technology that can be designed for the task in hand. A number of different tumour types could be embedded in a number of constructs that conform to different specifications, in terms of both the stroma and the nature and proportion of the supporting cells. Although we have not described it here, energy and nutrient availability can also be controlled in line with standard tissue engineering techniques.

### 4.6 Future directions

We believe that our 3-D in vitro tumour model provides a platform on which complexity can be bestowed. We have documented some of these complex parameters, including various cell types, matrix densities, tumour hypoxia and angiogenic response. There exists, however, considerable scope to improve upon this early work. We would encourage other groups to exploit their areas of expertise in other tumour types and/or other associated supporting cells to further reduce the gap between the events that we see within in vitro models of cancer and those that occur in vivo*.*

## 5 Conclusions

Our use of tissue engineering principles has allowed us to create a biomimetic model of malignant tumour and stroma with many of the key in vivo attributes. The unique and important attributes of our model include a dense ACM containing cancer epithelial cells surrounded by a non-dense collagen hydrogel containing fibroblasts and endothelial cells, mimicking the reactive stroma. This arrangement has conferred on our model oxygen depletion within the ACM, resulting in hypoxia and the production of VEGF. These two attributes of cancer biology are fertile areas for future therapies. Our model is likely to assist in the understanding of these processes, and may provide a useful model in which putative therapies can be tested.

## Appendix A Figures with essential colour discrimination

Certain figures in this article, particularly Figs. 1–5, are difficult to interpret in black and white. The full colour images can be found in the on-line version, at http://dx.doi.org/10.1016/j.actbio.2013.04.028.
